# Utilization of Secondary Raw Materials from Rice and Buckwheat Processing for the Production of Enriched Bread: Optimization of Formulation, Physicochemical and Organoleptic Properties, Structural and Mechanical Properties, and Microbiological Safety

**DOI:** 10.3390/foods13172678

**Published:** 2024-08-25

**Authors:** Madina Yessembek, Baltash Tarabayev, Mukhtarbek Kakimov, Bożena Gajdzik, Radosław Wolniak, Michał Bembenek

**Affiliations:** 1 The Department of Food Technology and Processing Products, S. Seifullin Kazakh Agrotechnical Research University, Zhenis Avenue 62, Astana 010011, Kazakhstan; yessembek.madina@gmail.com (M.Y.); muhtarbek@mail.ru (M.K.); 2 Department of Industrial Informatics, Silesian University of Technology, 44-100 Gliwice, Poland; 3 Faculty of Organization and Management, Silesian University of Technology, 44-100 Gliwice, Poland; rwolniak@polsl.pl; 4 Faculty of Mechanical Engineering and Robotics, AGH University of Krakow, A. Mickiewicza 30, 30-059 Krakow, Poland; bembenek@agh.edu.pl

**Keywords:** secondary raw materials, rice bran, buckwheat bran, bread, complex sourdough starter, fermented brew, microbiological safety, mold, potato disease

## Abstract

Pursuing enhanced nutritional value in bakery products through technological advancements and new recipes is a promising facet of the food industry. This study focuses on incorporating rice and buckwheat brans, additional raw materials rich in biologically active substances, into bakery products. Utilizing a second-order rotatable plan, optimal ratios were determined—5% rice bran and 10% buckwheat bran. The application of these brans influenced dough and bread quality, reducing sugar content by 5% in dry form and 29% in the fermented brew, potentially aiding in diabetes prevention and cholesterol control. Introducing brans, especially in fermented brew, positively impacted microbiological stability, reducing the risk of mold and potato disease. The developed bread technology using rice and buckwheat brans in fermented brew significantly increased nutritional value, satisfying adult daily protein needs by 31.2%, fats by 15%, and dietary fibers by 18.4%. This innovative approach ensures a sufficient intake of essential vitamins and minerals, showcasing a promising avenue for creating healthier and more nutritious bakery products.

## 1. Introduction

Most of the technologies used in the grain processing industry are mainly multiwaste. In recent years, much attention has been paid to the secondary raw materials that are generated during the processing of grain crops [1,2,3,4,5].

The secondary raw materials of grain crop processing include grain waste, bran [6,7], husk [8], and germ, but at the same time the listed wastes do not find proper practical application in the food industry. For example, when processing rice grain into groats, bran is produced [9,10], the yield of which is about 11%; when processing peas [11], the share of waste and by-products is 33%; and when processing buckwheat grain [12,13] into groats, the share of waste is not less than 26% of the total volume of processed grain.

When grain is processed into cereals, the anatomical parts of the grain, the aleurone layer and the germ, which are valuable to humans, are wasted together with the resulting bran [14]. Bran obtained in different peeling systems of various cereal crops has a high content of fiber, fat, and protein [15]. In addition, a high content of vitamins such as B1, B2, E, and PP has been observed. In some cultures, the content of vitamins in bran exceeds their content in grain more than three times [16,17].

According to experts’ calculations [18], the level of self-sufficiency of a country’s population in rice reaches 116–120%, and the volume of rice products averaged 141.1 to 173.9 thousand tons over the last 5 years. Kazakhstan can produce rice in the volume of 449.4–481.9 thousand tons, and the yield of rice bran when processing raw rice into groats is 10–15%.

Buckwheat and its products are known to have good digestibility and high nutritional value and are characterized by a low glycemic index [19,20].

When buckwheat grain is processed into groats, buckwheat bran is formed as a by-product. An important advantage of buckwheat bran is the complexity of its chemical composition. It contains a wide range of natural biologically active components, which, when added to food, have a beneficial effect on the human body [21,22].

Studies on the chemical composition of rice and buckwheat brans showed that they contain a fairly high amount of protein (11.8% and 23.82%), fat (12.01% and 4.95%), and carbohydrate (73.33% and 60.14%), respectively. The results of comparative analysis of chemical composition show that rice bran is characterized by a high content of fat 8 times more than in wheat flour of the first grade, and buckwheat bran surpasses wheat flour of the first grade in protein content 2.1 times and fat 3.5 times.

The sum of essential amino acids of rice and buckwheat brans is 3.76 and 9.29 g/100 g protein, respectively, which is 1.3–3.5 times higher than the content of first-grade wheat flour (2.7 g/100 g protein). Thus, the proteins of rice and buckwheat brans are characterized by a high content of essential and substitutable amino acids, many of which are superior to the proteins of first-grade wheat flour, which shows real prospects for their use in the composition of bakery products to increase biological value [23].

According to the literature data [24], buckwheat bran contains important representatives of sterols such as capesterol (211 μg/g) and β—sitosterol (1456 μg/g), which have immunomodulatory, oncoprotective, antioxidant, and hypoglycemic properties.

Thus, based on the above facts, we can conclude that the secondary raw material resources of grain crop processing represent a source of enrichment of food products. Its application in the baking industry is the most rational option.

The analysis of scientific and industrial developments indicates that currently the production of products of increased nutritional value with functional properties based on cereal crops is actively developing in the world [25,26,27]. Their functional effect is due to the presence of a whole complex of enriching ingredients (biologically active substances): dietary fiber, vitamins, minerals, proteins, lipids, antioxidants, prebiotic carbohydrates, etc. [28].

The conducted research on studying the influence of rice bran on the nutritional value of bakery products based on the assessment of quality and nutritional value of the finished product showed that the introduction of rice bran into the dough significantly affects the quality indicators of bread. It was found that the dosage of 15% to the mass of flour is the most optimal for obtaining a product with high organoleptic and physicochemical quality indicators [29].

Researchers [30] conducted studies on the effect of adding 2–20% of rice bran to wheat flour on the rheological properties of dough using a farinograph, consistograph, and alveograph. The changes in physicochemical properties of the dough were significant even after the addition of 20% bran. The addition of rice bran to wheat flour had a negative effect on the composition of the mixture, resulting in lower water absorption capacity and lower dough quality. In general, the organoleptic characteristics of the product, such as color, odor, and taste, decreased with increasing dosage of bran. The conducted studies allowed us to establish the maximum permissible dosage of rice bran in the production of bakery products, which is 10%.

The possibility of using buckwheat bran in bakery product formulations has been investigated. It was found that the addition of bran increases the antioxidant activity of bread by 12.5%, the content of dietary fiber by 21.0%, and mineral content by 15.0–39.5%. As a result, the total cost of the product decreased by 44.5%, and the developed bread can expand the range of functional food products and provide a waste-free technology of grain crop processing [31].

Our author’s composition [32] investigated the influence of secondary raw materials of grain crop processing on the rheological properties of dough. The main objects of research were flour composite mixtures obtained by mixing first-grade wheat baking flour, rice bran, and buckwheat bran in the percentage ratio of 95:2:3, 90:3:7, 85:4:11, 80:5:15, and 75:6:19, respectively. According to the alviogram, the baking ability (W) starts to decrease compared to that of the control from the ratio of wheat flour, rice bran, and buckwheat bran—85:4:11, respectively. Resistance of dough to deformation increased according to the ratio of wheat flour, rice bran, and buckwheat bran—80:5:15, respectively. The analysis of dough rheological indices according to the data of the Mixolab 2 device allowed us to estimate the properties of grain raw materials and to predict the quality of the finished product with a high degree of reliability. According to the results of profiler indexes analysis, it was found that with an increasing dosage of rice bran and buckwheat bran indexes of water absorption capacity increase, which allows us to increase the dough yield. Starting from the ratio of wheat flour, rice bran, and buckwheat bran—85:4:11—a decrease in the index of kneading was observed. Based on the results of the study, it was found that the introduction of secondary raw materials of cereal crops—rice and buckwheat brans in the formulation of wheat bread in the dosage of 4–11%, respectively—improves the rheological and physicochemical properties of dough, which will contribute to the creation of a new type of enriched bread.

A significant theoretical and practical contribution to the development and improvement of bread technology using rice and buckwheat brans was made by Tolstoguzov et al. [23,31,33,34,35,36,37,38,39].

Currently, a serious problem of bakery products is their vulnerability to microbial spoilage under the influence of bacteria and mold fungi, as it not only leads to deterioration of the appearance of bakery products but also negatively affects their quality and safety [40,41,42].

The most dangerous and widespread disease of bread is potato disease, which is often observed in regions with a hot climate and summertime. In recent years, due to the widespread use of packaging in polymeric materials, which is a provoking factor for the development of microbial spoilage, the spread of cases of potato disease is noted not only in the southern regions, but also in the northern regions of Kazakhstan and the spring–summer period and in winter [43].

Thus, numerous studies have been conducted on the use of rice and buckwheat brans for the enrichment of bakery products. However, studies on the inclusion of a complex herbal supplement consisting of rice and buckwheat brans in the composition of bakery products have not been sufficiently investigated. In addition, there are no measures to prevent potato bread disease and prevent bread mold, which is one of the most important aspects of bakery product production. From this point of view, this present study aims to develop a bread technology based on rice and buckwheat brans with increased nutritional value and microbiological safety.

## 2. Materials and Methods

### 2.1. Materials

The main and additional raw materials were used: wheat baking flour of the first grade, produced in “Tsesna-Astyk” LLP (Astana, Kazakhstan); rice bran, produced by one of the dynamically developing companies of the agricultural industry, in PT “Abzal and Company” (Kyzylorda region, Kazakhstan); buckwheat bran, selected from “Yeger” LLP (Pavlodar region, Kazakhstan). Granulated sugar (TSZ, Taraz, Kazakhstan), table salt (Aral-Tuz JSC, Almaty, Kazakhstan), pressed yeast (Saf-Neva LLC, Voronezh region, Russia), unfermented barley malt (Leipurin LLP, Almaty, Kazakhstan) were purchased from a local supermarket in Astana, Kazakhstan. As pure cultures of lactic acid bacteria (LAB), the used strains were *Lactiplantibacillus plantarum* 1, *Levilactobacillus brevis* E 120, as well as pure culture of yeast *Saccharomyces cerevisiae* st. L-1 (aqueous suspension) from the museum collection of the St. Petersburg branch of the Research Institute of Baking Industry.

### 2.2. Mathematical Modeling and Optimization of Recipe Composition of Bakery Products Using Rice and Buckwheat Brans

To obtain a mathematical model for optimizing the recipe composition of bakery products using secondary raw materials of grain crop processing, which is a regression equation, a rotatable plan of the second order (Box plan) was used. When the number of factors x was equal to 3, and the number of experiments more than 20, the number of experiments at the zero point was 6 and the number of equation coefficients—10.

The baking strength of dough (Y1), water absorption capacity (WAC) (Y2), and organoleptic indices (Y3) of bread using recycled grain processing raw materials are affected by the following factors: addition of wheat flour (W, g), addition of rice bran (R, g), addition of buckwheat bran (B, g).

The above factors determine specific production conditions, so it is reasonable to adjust the system of regression equations to these factors.

The regression equation has the following form (Equation (1)):

y_1_ = b_0_ + b_1_x_1_ + b_2_x_2_ + b_3_x_3_ + b_12_x_1_x_2_ + b_13_x_3_ + b_23_x_2_x_3_ + b_11_x_12_ + b_22_x_22_ + b_33_x_23_ (1)

The coding of intervals and levels of variation in input factors of optimization of the composition of bakery products using secondary raw materials of grain crop processing are presented in Table 1.

The experimental part was carried out according to the made planning matrix. The planning matrix of experiments is presented in Table 2.

### 2.3. Breadmaking Process

Two methods of dough preparation were studied: the straight dough method [44] and a complex sourdough starter [45,46] consisting of rice and buckwheat brans. The control was wheat bread prepared from first-grade wheat baking flour with dough moisture of 47.0% without adding rice and buckwheat brans.

**Figure 1:**
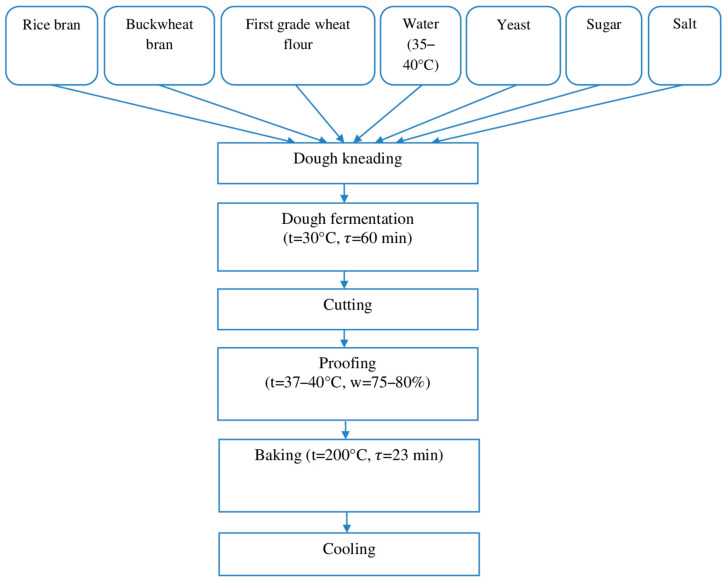
Technological scheme of dough preparation by straight dough method.

In the straight dough method, 5% rice bran and 10% buckwheat bran in dry form and 85% of first-grade wheat baking flour, salt, and pressed baking yeast, as well as sugar according to the recipe and water in the amount that provides dough moisture 48.0%, were added to the dough (Figure 1).

The peculiarity of the preparation of dough on complex sourdough starter is the use of complex sourdough starter, by fermentation of sugared brew at a temperature of 32–34 °C, as well as using a composition of pure cultures of starter microorganisms. These compositions have probiotic properties and antibiotic action against spore microflora. At the same time, traditional biological leaven (thick, liquid without brewing, liquid with brewing) is excluded from the technological process, and the amount of flour introduced in brewed form is increased (25–35%).

The sourdough was prepared on pure cultures of microorganisms in liquid form by the “Collection of technological instructions for the production of bakery products” [47].

For preparation of complex sourdough starter, pure cultures of lactic acid bacteria *L. plantarum* 1, *L. brevis* E 120 (1.5 mL each), and yeast *S. cerevisiae* st. L-1 (1 mL of aqueous suspension) were used.

Preparation of complex sourdough starter includes dilution and production cycles.

The dilution cycle was used with a small mass of sourdough starter (500 g in the first phase). Microbial cultures were fused together, added to the saccharified brew of rice and buckwheat brans with the addition of unfermented barley malt (2% to the weight of flours) and water, and left to ferment for 20 h.

The complex sourdough starter bred by breeding cycle using pure cultures of lactic acid bacteria and starter yeast in liquid form in the production cycle was maintained by refreshing with a sugared brew in the ratio of 1:1–1:9, i.e., complex sourdough starter—nutrition (sugared brew) depending on the duration of sourdough starter fermentation.

Eighty percent (80%) of the complex sourdough starter obtained was used for bread production and the remaining part (20%) was used for its refreshing.

**Figure 2:**
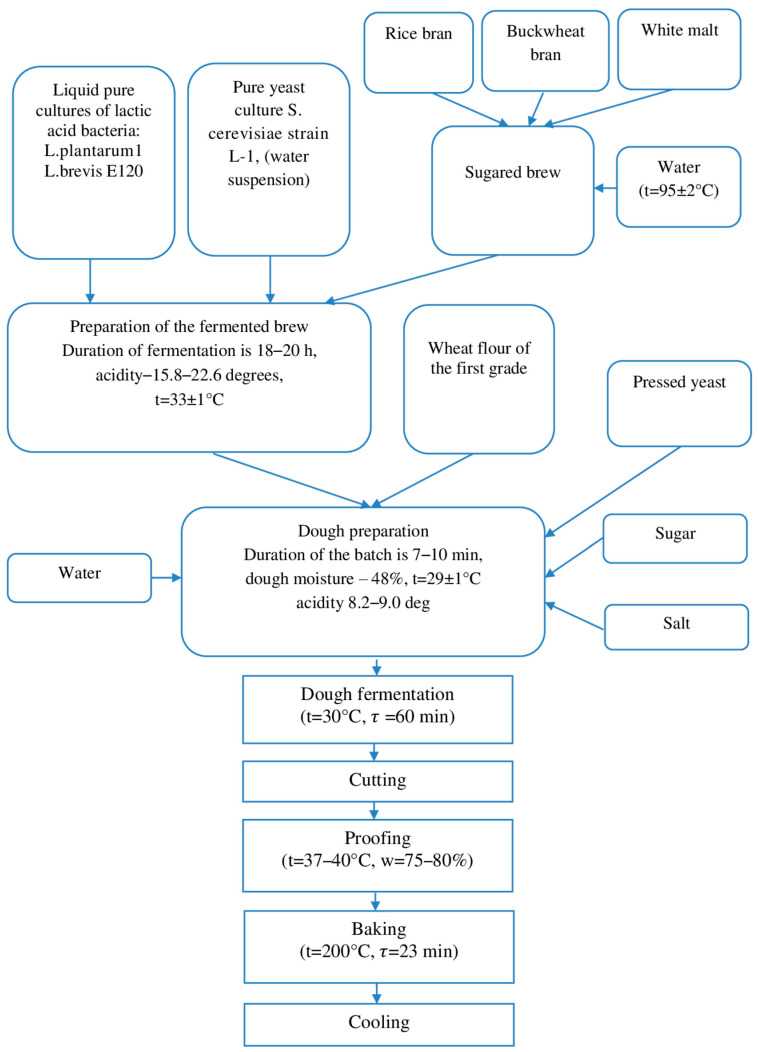
Technological scheme of dough preparation on complex starter culture.

The technological scheme of dough preparation on complex sourdough starter is presented in Figure 2.

### 2.4. Characterization of Doughs

#### 2.4.1. Determination of Dough Rheological Properties

The baking strength of the dough was determined on an alveograph (Alveolab, CHOPIN Technologies, Villeneuve-la-Garenne, France) [48]. The water solubility of the dough on the device Mixolab 2 (CHOPIN Technologies, France) was evaluated according to the protocol “Chopin+”, which assumes 5 temperature intervals at which the study is carried out [49].

#### 2.4.2. Determination of Physical and Chemical Parameters of the Dough

Dough moisture was determined by the express method by drying on the device for moisture determination VNIIHP-VCh [50]. Acidity—according to GOST 5898-2022 “Confectionery. Methods for determination of acidity and alkalinity” [51] by titration method in the presence of phenolphthalein with sodium hydroxide solution and expressed in degrees. Volume increase—according to GOST 27669-88 “Wheat bread flour. Method for experimental laboratory breadmaking” [52] by the method of trial laboratory baking of bread. Dough lifting force was determined according to GOST 171-2015 “Pressed baking yeast. Technical conditions” [53] by the balloon method. 

### 2.5. Characterization of Bread

#### 2.5.1. Determination of Physicochemical Parameters

Physicochemical parameters of bread were determined 3 h after baking. The moisture content of crumb and whole bread was determined according to GOST 21094-2022 “Bakery products. Methods of the moisture determination” [54] gravimetric method by drying a sample of bread in a desiccator. The acidity of finished products was determined by GOST 5670-96 “Bread, rolls and buns. Methods for determination of acidity” [55] by the arbitration method: being titrated with sodium hydroxide solution in the presence of phenolphthalein and expressed in degrees. Bread porosity was determined by the Zavyalov method according to GOST 5669-96 “Bakery products. Method for determination of porosity” [56] and expressed in percent. Specific volume was estimated according to a widely used method [50] and expressed in cm^3^/g. Alcohol content according to GOST 5962-2013 “Rectified ethyl alcohol from food raw materials. Technical conditions” [57]. The content of volatile acids was demonstrated by the method of distillation of volatile acids and titration of the resulting distillation of 0.05 mol/dm^3^ was carried out with sodium or potassium hydroxide solution [58]. The mass fraction of sugar was determined by the Bertrand method according to GOST 5672-2022 “Bakery products. Methods for determination of sugar content” [59].

#### 2.5.2. Determination of Organoleptic Parameters

In total, 10 researchers from the St. Petersburg branch of the Research Institute of the Bakery Industry took part in the assessment of the organoleptic parameters of bread. Three variants of experimental bread samples were presented for tasting. Experimental samples were made with the introduction of rice and buckwheat brans in dry form and in the form of fermented brew. As a control sample, wheat bread made from first-grade wheat baking flour was presented. During the tasting, the bakery products were evaluated according to a 5-point food evaluation system.

To determine the organoleptic characteristics of bakery products, we were guided by the method described in GOST 5667-2022, “Bakery products. Acceptance rules, sampling methods, methods for determining organoleptic parameters and mass of products” [60]. Appearance (shape, surface, and color), the presence of foreign inclusions, signs of disease, and mold products were determined by inspection of products under natural light. To determine the condition of the crumb, samples were cut along the width close to the middle of the product. The quality of being baked was determined by lightly pressing the crumb surface in the center of the product with fingers. The thickness and porosity of the crumb were determined visually under natural light. In determining the taste, crunch was determined from mineral impurities’ part of the sample weighing 2–3 g chewed for 5–10 s and compared with the requirements of the standardization document and technical document. Odor was determined by two- or three-time deep inhalation of air through the nose from the surface of the whole product. Then, the product was cut in the middle and the procedure was repeated by inhaling air near the crumb of the product.

#### 2.5.3. Determination of Structural–Mechanical Properties

To study the structural–mechanical properties of bread crumbs during storage, we investigated such indicators as moisture, crumbliness, and physical properties of crumb: hardness index. The studies were conducted 2 h after baking, when the temperature in the center of the crumb was 30–36 °C. Then, they were packed in perforated polyethylene bags and stored at room temperature. Samples were analyzed after 24, 48, and 72 h. The crumbliness of bread was determined according to the generally accepted method [61]. For physical properties of the crumb, the hardness index was determined on an ST-2 structrometer.

#### 2.5.4. Microbiological Safety

To determine the possibility of suppressing the potato disease of bread, the method of putting finished products infected with spore bacteria into conditions provoking the development of *Bacillus subtilis* was used. To create provoking conditions, dry bread crumbs infected with *B. subtilis* spore-forming bacteria and containing 108 KOE/g of spores were introduced during dough kneading. To determine the content of *B. subtilis* spores in the infected crumb, the method described in the book *Microbiology of Baking Production* (Afanasyeva O.V., 2003) was used [62]. The bread was stored according to the instructions for the prevention of potato bread disease [63], for which, immediately after baking, the bread was wrapped in damp paper and placed in a thermostat at 37 °C. After 24 h, the bread was cut with a knife and the presence of signs of disease (specific unpleasant odor, sticky crumb) was determined visually. In the absence of disease, the bread was further incubated under the same conditions.

To establish the influence of bread preparation technology on the rate of mold development, model experiments were conducted with infection of sterile bread slices with a pure culture of mold fungi *Penicillium chrysogenum*.

Strains of baking pests causing bread diseases were obtained from the collection of microorganisms “Lactic acid bacteria and yeasts for the baking industry” in the St. Petersburg branch of FGANU NIIHP.

#### 2.5.5. Chemical Composition and Nutritional Value of Enriched Bread

In bread with the use of rice and buckwheat brans in the form of fermented brew, we determined the chemical composition: proteins—according to GOST 10846-91 “Grain and products of its processing. Method for determination of protein” [64]; fats—according to GOST 5668-2022 “Bakery products. Methods for determination of fat content” [65]; carbohydrates—according to GOST R 51636-2000 “Fodder mixed fodder and animal feed raw stuffs. Photometric with 2,4-dinitrophenol and permanganate methods for determination of water soluble carbohydrates” [66]; dietary fiber—according to GOST 31675-2012 “Feeds. Methods for determination of crude fibre content with intermediate filtration” [67]; vitamins—according to GOST R 54634-2011 “Functional food products. Method of vitamin E determination” [68]; mineral elements—according to GOST 32343-2013 “Feed, compound feed. Determination of the content of calcium, copper, iron, magnesium, manganese, potassium, sodium and zinc by atomic absorption spectrometry” [69]. In finished products, the calculation of chemical composition, energy value, and the degree of coverage of the daily requirement in substances was carried out using the program “COMPLEX”.

### 2.6. Statistical Analysis of the Data

All of the experiments were carried out a total of five times. Statistical analysis was performed using Excel 2019 (17) software. Comparison of the influence of factors was carried out by the method with significance tested at the 95% confidence level, and differences among means were determined using the least significant difference and Duncan’s test of two-factor analysis of variance with one repetition (ANOVA). The confidence intervals shown in the histograms and in the table reflect the accuracy of the used methods.

## 3. Results

### 3.1. Optimization of Recipe Composition of Bakery Products Using Rice and Buckwheat Brans

Studies on achieving the best value of rheological parameters of dough for bakery products using rice and buckwheat brans have been carried out. Several experimental studies were carried out to determine the rheological parameters with different ratios of input parameters x. The results are summarized in Table 3.

Table 3 shows that 20 experiments were conducted, of which there were 15 with different flour-to-bran ratios and 5 experiments at the zero point of x values. The divergence of the results at the zero point was ±1.

**Figure 3:**
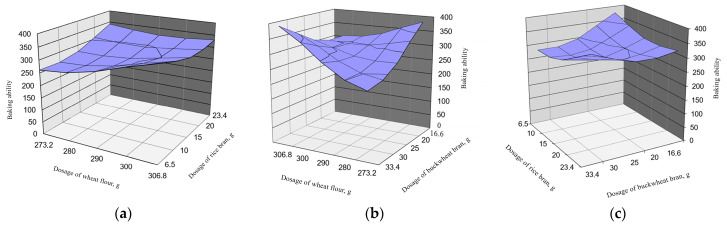
Dependence of flour and bran addition on baking ability: (**a**) dependence of wheat flour and rice bran addition; (**b**) dependence of wheat flour and buckwheat bran addition; (**c**) dependence of rice bran and buckwheat bran addition.

Optimization of baking strength of dough composition bakery products with the use of secondary raw materials of grain crop processing is presented in Figure 3.

Figure 3 shows that the rheology values decrease with decreasing wheat flour concentration and increasing total bran content. However, with a slight decrease in wheat flour content and the addition of rice and buckwheat brans, the desired rheology value can be achieved.

It is easy to see that the planning matrix is orthogonal with linearly independent vector columns; hence, the diagonality of the matrix and the system of equations is normal, and hence the mutual independence of the estimates of the coefficients of the regression equation is shown. Then, the regression equation of optimization of the composition of bakery products using secondary raw materials of grain crop processing with the best efficiency coefficient Y_1_ has the form as follows (Equation (2)):

Y_1_ = 279.2727848 − 13.7001x_1_ − 6.97742x_2_ + 8.851344x_3_ − 9.875x_1_x_2_ + 21.625x_3_ + 11.875x_2_x_3_ + 5.835886x_12_ + 9.89308x_22_ + 10.7751x_23_ (2)

**Figure 4:**
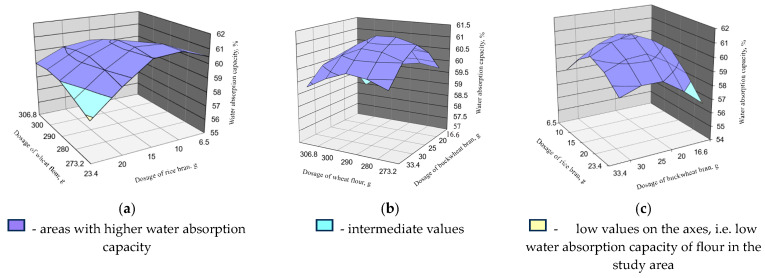
Dependence of flour and bran addition on water absorption capacity: (**a**) dependence of wheat flour and rice bran addition; (**b**) dependence of wheat flour and buckwheat bran addition; (**c**) dependence of rice bran and buckwheat bran addition.

Optimization by water absorption capacity of flour composition of bakery products with the use of secondary raw materials of grain crop processing is presented in Figure 4.

Figure 4 shows that the water absorption capacity of flour increases as the content of rice and buckwheat brans decreases. Thus, by slightly decreasing the content of rice and buckwheat brans and increasing the content of wheat flour, the required value of the water absorption capacity of flour can be achieved.

The regression equation for optimizing the composition of bakery products using secondary raw materials of grain crop processing with the best efficiency coefficient Y_2_ is as follows (Equation (3)):

Y_2_ = 61.18210125 + 0.226042x_1_ + 0.208474x_2_ − 0.14347x_3_ + 0.35x_1_x_2_ + 0.075x_3_ + 0.2x_2_x_3_ + 0.20386x_12_ − 0.64486x_22_
− 0.46846x_23_ (3)

**Figure 5:**
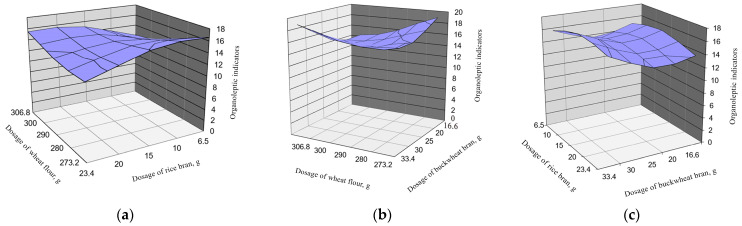
Dependence of flour and bran addition on organoleptic parameters: (**a**) dependence of wheat flour and rice bran addition; (**b**) dependence of wheat flour and buckwheat bran addition; (**c**) dependence of rice bran and buckwheat bran addition.

Optimization by organoleptic indicators of the composition of bakery products with the use of secondary raw materials of grain crop processing is presented in Figure 5.

Figure 5 shows that the organoleptic parameters significantly depend on the content of rice bran due to the higher fat content in its composition.

Thus, the optimum composition for obtaining bakery products with the addition of secondary raw materials processing grain crops falls on the point where the addition of wheat flour is 280 g and rice bran 14 g, with the addition of 28 g of buckwheat bran, and at this point quality indicators of the baking strength of dough are 350, water absorption capacity is 61.1%, and evaluation of organoleptic indicators is 18 points. The addition percentage of wheat flour is 85%, rice bran 5%, and buckwheat bran 10%.

On the basis of optimization of the recipe composition, it was determined that the maximum value of the complex quality index of a new type of bread enriched with rice and buckwheat brans is observed with the addition of rice bran 5% and buckwheat bran 10%. A further increase in the bread formulation of these components leads to a decrease in its rheological properties, which may be caused by mechanical destruction of both gluten backbone and structural elements of swollen dough proteins. Rheological properties of dough as integral indices describing the state of dough during kneading during the whole technological process allow us with a high degree of reliability to assess the properties of grain raw materials and predict the quality of the finished product. And deterioration of the organoleptic indicators of bread quality is associated with the chemical composition of bran, especially because of the high fat content in rice bran [23].

Comparing the results obtained with the results of Boldina et al. [29], it can be seen that according to the results of the mathematical processing of experimental data, the optimal dose of rice bran was 15% of the flour weight. And as a result of the research conducted by Saeed et al. [30], the optimum dose of rice bran similar to our result was 10%. The study by Nikiforova et al. [24] also established the maximum allowable dosage of buckwheat bran as 30% to the weight of flour. The comparative analysis shows that our results do not exceed the results of other authors and that the optimal dosages established by us can contribute to obtaining bread of higher quality.

### 3.2. Dough Properties

To study the influence of the rice and buckwheat bran addition method on the dough and bread quality indicators, dough for experimental samples was prepared by the straight dough method and on the complex sourdough starter with an optimal ratio of rice and buckwheat brans being 5:10, respectively. The control was wheat bread prepared from first-grade wheat baking flour with dough moisture of 47.0% without the addition of rice and buckwheat brans. The technological parameters of dough preparation are given in Table 4.

The results of studies showed that the introduction of rice and buckwheat brans in dry form and the form of fermented brew promotes acid accumulation in the dough.

Experimentally, it was revealed that the introduction of rice and buckwheat brans in dry form and the form of fermented brew increases the volume of dough 1.8–2 times and in the control sample only 1.5 times.

When using rice and buckwheat brans in the form of fermented brew, the duration of proofing was reduced by 19%, while in the case of dry addition, it remained unchanged compared to that of the control.

The existing regularities of changes in dough properties are explained by hydrolytic processes during dough ripening, which leads to greater pliability of the dough gluten skeleton to stretching under the action of formed carbon dioxide bubbles in the process of alcoholic fermentation. The obtained research results were considered in comparison with the results obtained using rice and buckwheat brans [70,71].

### 3.3. Physicochemical and Sensory Properties of Bread

Finished products were evaluated by physicochemical and organoleptic parameters. Analysis of the obtained results, presented in Table 5, showed that the introduction of rice and buckwheat brans in dry form and the form of fermented brew increased acidity by 1.5–3.3 times, respectively, and this is due to the high intensity of biotechnological processes occurring during the ripening of semi-finished products, as well as the accumulation of organic acids and carbon dioxide. 

The content of alcohol and volatile acids increased by 2–2.9 times when rice and buckwheat brans were introduced as fermented brew, respectively.

At the introduction of rice and buckwheat brans in dry form, the porosity and specific volume of bakery products did not differ from the control sample, while when added in the form of fermented brew, they decreased by 7.6% and 15.2%, respectively.

Reduction in sugar content in bread when adding rice and buckwheat brans in dry form was by 5%, and in the form of fermented brew by 29%, which reduces the risk of diabetes mellitus and helps to reduce cholesterol and blood sugar.

Organoleptic parameters of the samples under study were evaluated 24 h after baking by the following indicators: shape, surface condition, product color, crumb condition, taste, and smell.

**Figure 6:**
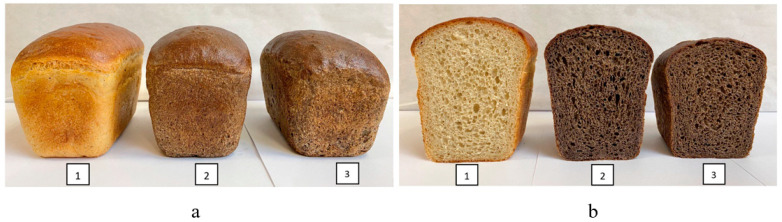
Bread samples: (**a**) appearance of bread samples; (**b**) crumb condition of bread samples; 1—control (without additives); 2—bread with the addition of rice and buckwheat brans in dry form; 3—bread with the addition of rice and buckwheat brans in the form of fermented brew.

As a result, the obtained bread samples had a regular shape, as well as a smooth and glossy surface. The color of the products was uniform, with a brownish-grey tint (Figure 6a).

The crumb of experimental bread samples was characterized by higher elasticity and softness. The porosity of the samples was uniform and thin-walled (Figure 6b).

In bread with the addition of rice and buckwheat brans in the form of fermented brew, the sourness was felt in the taste, and the smell was pleasant, peculiar to the additive, while in bread with rice and buckwheat brans in dry form, the taste was bland and the smell was unexpressed.

On the basis of the obtained results, it can be concluded that during the production of bread from wheat flour of the first grade the introduction of rice and buckwheat brans in the form of fermented brew contributes to the formation of improved organoleptic quality indicators of finished products, while improving the state of the product surface, crumb structure, flavor, and aroma. It can be assumed that the improvement of taste qualities of wheat flour products with the introduction of rice and buckwheat brans in the form of fermented brew is conditioned by the technology of its preparation. The research results are consistent with the results obtained by Shansharova et al. [70].

### 3.4. Structural and Mechanical Properties of Bread

The purpose of this stage of research was to study changes in the storage process of moisture content and structural and mechanical properties (crumbliness and physical properties of crumb: hardness index) of bread crumbs made with the use of rice and buckwheat brans in the ratio of 5:10 in dry form and the form of fermented brew.

Studies on the influence of the method of introduction of rice and buckwheat brans on the preservation of freshness of bread after 24, 48, and 72 h of storage and packed after cooling to a temperature of 30–36 °C (in the center of the crumb) in perforated polyethylene bags were carried out.

**Figure 7:**
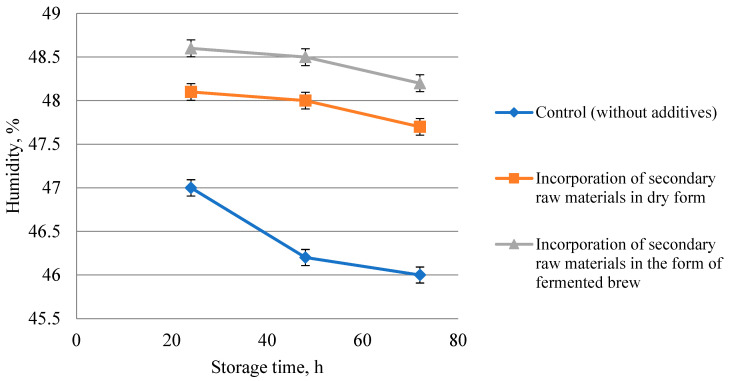
Influence of rice and buckwheat bran application method on the change in crumb mass fraction of moisture during bread storage.

**Figure 8:**
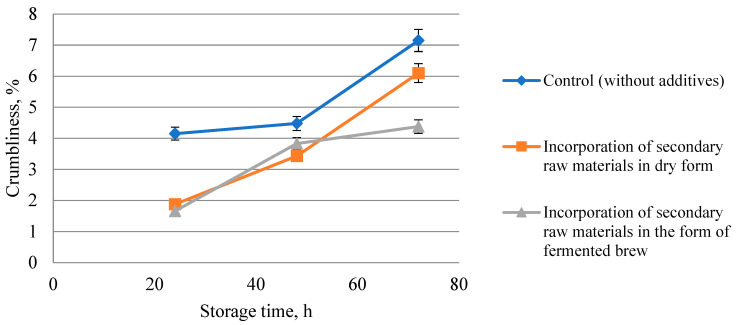
Influence of rice and buckwheat bran application method on bread crumbliness in the storage process.

**Figure 9:**
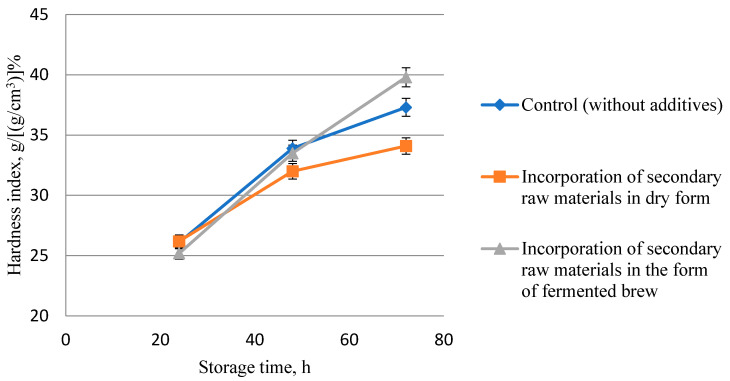
Effect of rice and buckwheat bran application method on bread crumb hardness index in the storage process.

The results of the research are presented in Table 6 and Figure 7, Figure 8 and Figure 9.

Analysis of research results showed that during 24–72 h crumb moisture in all bread samples decreased, and the most intensive process of moisture loss was observed in control bread during 24–48 h (Figure 7).

As a result of the research, the crumbliness of bread crumbs during storage increased in all bread samples (Figure 8). However, the control bread differed from all samples by a higher value of crumbliness during the whole storage period (72 h). This proves that bread using rice and buckwheat brans can provide an increase in shelf life.

During storage from 48 to 72 h, the lowest value of crumbliness was observed in bread samples with rice and buckwheat brans in the form of fermented brew, while in other samples the intensity of staling processes was high.

As a result of this study, it was revealed that there was a decrease in the index of crumb hardness index during storage of all bread samples, regardless of the methods of introducing secondary raw materials of grain crop processing (Figure 9).

It should be noted that during storage from 48 to 72 h in the samples of bread enriched with rice and buckwheat brans in dry form, an unpleasant specific odor appeared.

Due to the inclusion of rice and buckwheat brans in the composition of bread, structural and mechanical parameters are improved during storage of all bread samples, regardless of the ways of introducing secondary raw materials of grain crop processing. It is proved that during storage of bakery products, along with cooling, the process of stale begins. The process is accompanied by a change in the state of the crumb, which from soft consistency goes to hard. The more stale the bread, the more it crumbles. It was found that the crumbliness of the crumb of the control sample of bread increased from 4.15 to 7.15%. In bread samples using secondary products, it increased from 1.88 to 6.1% in dry form, and from 1.66 to 4.38% in the form of fermented brew. This proves that bread using secondary products provides an increase in its shelf life. A similar result was observed in the studies of Nikiforova et al. [24], when buckwheat bran was added to wheat bread formulation. However, it should be noted that in the process of storage from 48 to 72 h in the samples of bread enriched with rice and buckwheat brans in dry form, an unpleasant specific odor appeared. 

### 3.5. Microbiological Safety of Bread

It is known that for bakery products the degree of microbial contamination of the main baking raw materials has a significant impact on the development of both potato disease and mold growth.

When preparing bread using rice and buckwheat brans in the traditional way, it was found that the use of secondary raw materials in dry form provokes the growth of mold fungi in the process of bread storage compared to products prepared on complex sourdough starter and without the addition of secondary raw materials.

**Figure 10:**
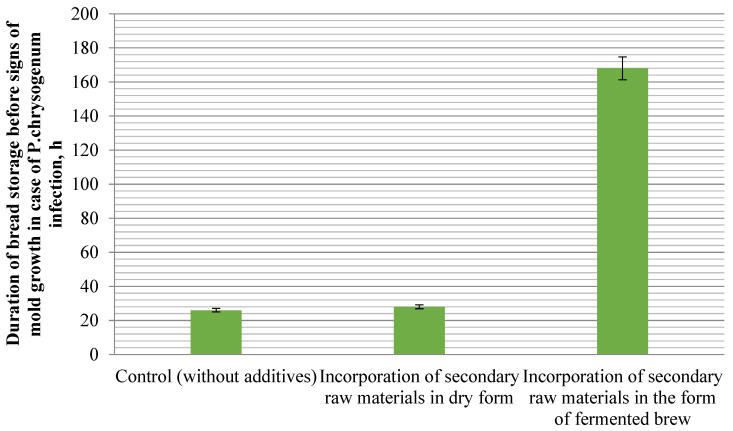
Effect of rice and buckwheat bran application method on bread resistance to moldiness.

Upon the infection of sterile bread slices with rice and buckwheat brans on the complex sourdough starter, the pure culture of the mold Penicillium chrysogenum, mold spores did not appear, which proves that the use of secondary raw materials in the form of fermented brew during dough kneading provides its microbiological purity, especially the resistance of bread to mold growth. At the same time, on the surface of bread slices prepared with rice and buckwheat brans in dry form, mold growth was observed after 26 h. While on a slice of control bread, prepared from first-grade wheat flour without the addition of secondary raw materials, mold spores appeared after 28 h (Figure 10).

Considering that the biological method involving the use of acidifying components is one of the most effective ways to protect bread from potato blight, the effect of rice and buckwheat brans on the suppression of potato bacillus spores was investigated.

**Figure 11:**
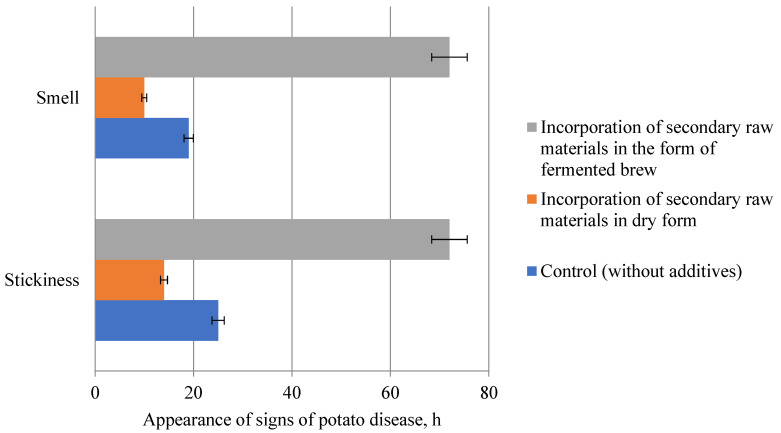
Effect of rice and buckwheat bran application method on resistance to potato bread disease.

As a result of trial baking, it was found that the appearance of signs of potato disease after 10 h was in the form of an unpleasant odor, and after 14 h the stickiness of the crumb was observed in the sample of bread prepared with the addition of rice and buckwheat brans in dry form. In the control sample of bread prepared from first-grade wheat flour without the addition of secondary raw materials, an unpleasant odor and stickiness of the crumb appeared after 19 and 25 h, respectively. The bread sample with the addition of rice and buckwheat brans in the form of fermented brew did not get potato disease (Figure 11).

As a result of the conducted research, it was found that the introduction of rice and buckwheat brans in the form of fermented brew increases antagonistic activity to *B. subtilis* and completely suppresses the development of spores of potato bacillus.

According to the results of studies of microbiological indicators of bread quality, it was found that the application of secondary products of cereal crops, rice and buckwheat brans in the form of fermented brew, increases the resistance of bread to mold and completely suppresses the development of potato disease. This indicates that the application of secondary products of cereal crops in the form of fermented brew has a significant effect on microbial spoilage of bread, compared to traditional methods of bread preparation.

The technology of complex sourdough starter is characterized by the use of pure cultures of starter microorganisms with probiotic properties and antagonistic activity to spore microflora in the breeding cycle. Selected strains of lactic acid bacteria (*L. plantarum* 1, *L. brevis* E 120) produce lactocins that inhibit the development of both bacteria and mold fungi in finished products. The research results are consistent with the results obtained by Savkina et al. [72].

### 3.6. Study of Chemical Composition and Nutritional Value of Enriched Bread

The conducted studies and the results obtained during the experiments allowed us to develop a technology of bread with the use of rice and buckwheat brans in the form of fermented brew, which showed the best quality indicators.

In this regard, a comparative assessment of the chemical composition and nutritional value of bread was carried out in the control variant and bread with the use of rice and buckwheat brans in the form of fermented brew (Table 7).

Based on the conducted research, it was established that the introduction of rice and buckwheat brans into the bread formulation in the form of fermented brew led to an increase in the content of protein by 1.5 times, fat by 6 times, iron by 475.8%, magnesium by 504.3%, calcium by 74.1%, potassium by 183.5%, and phosphorus by 224%, and this also allowed us to enrich the bread with B vitamins.

At the next stage of research, the degree of satisfaction with food substances at consumption of bread with the use of rice and buckwheat brans in the form of fermented brew was determined (Table 8) by the “Norms of physiological requirements in energy and food substances for different population groups of the Republic of Kazakhstan” [73].

Table 8 shows the consumption of bread with the use of rice and buckwheat brans in the form of fermented brew. In comparison with the control sample, it allows us to satisfy the daily requirement of an adult in proteins by 31.2%, fats by 15%, dietary fiber by 18.4%, as well as to provide sufficient intake of vitamins and minerals into the body.

Due to the inclusion of rice and buckwheat brans in the composition of bread, the nutritional value of ready bread increases. This is confirmed by the comparative chemical analysis of the developed bread in comparison with the control sample (Table 6). Due to the chemical composition, namely the content of protein, fat, fiber, vitamins, and minerals [23], rice and buckwheat brans have high antioxidant activity, which is manifested in the ready bread. This indicates the prospect of developing and introducing to the consumer market a new bread with increased nutritional value. Comparing the results with the research work of Boldina et al., it can be seen that the energy value of the bread developed by us is higher and the indices meet all standards [29].

## 4. Conclusions

Based on the optimization of the recipe composition, it was determined that the maximum value of the complex quality index of a new type of bread enriched with rice and buckwheat brans is observed with the addition of rice bran 5% and buckwheat bran 10%. A further increase in the bread formulation of these components leads to a decrease in its rheological properties, which may be caused by the mechanical destruction of both the gluten backbone and structural elements of swollen dough proteins.

It has been experimentally confirmed that preparation of dough from rice and buckwheat brans by the straight dough method and on complex sourdough starter improves physicochemical (alcohol content by 0.03–0.5%, volatile acid content by 0.1–1.4 times, and acidity by 1.5–3.3 times, respectively) and organoleptic quality indicators of finished products. The reduction in sugar content in bread at the introduction of rice and buckwheat brans in dry form by 5% and in the form of fermented brew by 29% was noted.

According to the results of studies of microbiological indicators of bread quality, it was found that the application of secondary products in the form of fermented brew increases the resistance of bread to mold and completely suppresses the development of potato disease. This indicates that the introduction of secondary products of cereal crops in the form of fermented brew has a significant effect on microbial spoilage of bread compared to traditional methods of bread preparation.

Due to the inclusion of rice and buckwheat brans in the composition of bread, the nutritional value of ready bread increases. This is confirmed by a comparative chemical analysis of the developed bread in comparison with the control sample. Due to the chemical composition, namely the content of protein, fat, fiber, vitamins, and minerals, rice and buckwheat brans have high antioxidant activity, which is manifested in the finished bread. This indicates the prospect of developing and introducing to the consumer market a new bread with increased nutritional value. 

## 5. Patents

1. Esembek Madina Zhanibekkyzy. Tarabaev Baltash Karimovich. Kuznetsova Lina Ivanovna. Omaraliyeva Aigul Makhmutovna. Botbayeva Zhanar Turlybekovna. Method of preparing a leaven for producing bread. For utility model No. 7716. filed 30 October 2022 and issued on 06 January 2023.
2. Esembek Madina Zhanibekkyzy. Tarabaev Baltash Karimovich. Kuznetsova Lina Ivanovna. Omaraliyeva Aigul Makhmutovna. Botbayeva Zhanar Turlybekovna. Brewed wheat bread production method. For utility model No. 7736. filed on 16 November 2022 and issued on 13 January 2023.

## Figures and Tables

**Table 1:** Coding of intervals and levels of variation in input factors.

Factors		Levels of Variation					Variation Intervals
Natural	Encoding	−1.68	−1	0	+1	+1.68	
W, g	x_1_	273.2	280	290	300	306.8	10
R, g	x_2_	6.5	10	15	20	23.4	5
B, g	x_3_	16.6	20	25	30	33.4	5

Abbreviations: W, wheat flour; R, rice bran; B, buckwheat bran.

**Table 2:** Rotatable planning matrix of experimental studies.

No.	Encoded Values			Natural Values			Experimental Values		
	x_1_	x_2_	x_3_	W, g	R, g	B, g	Y_1_	Y_2_	Y_3_
1	-	-	-	280	10	20			
2	-	-	+	280	10	30			
3	-	+	-	280	20	20			
4	-	+	+	280	20	30			
5	+	-	-	300	10	20			
6	+	-	+	300	10	30			
7	+	+	-	300	20	20			
8	+	+	+	300	20	30			
9	−1.68	0	0	306.82	15	25			
10	+1.68	0	0	273.18	15	25			
11	0	−1.68	0	290	23.41	25			
12	0	+1.68	0	290	6.59	25			
13	0	0	−1.68	290	15	33.41			
14	0	0	+1.68	290	15	16.59			
15	0	0	0	290	15	25			
16	0	0	0	290	15	25			
17	0	0	0	290	15	25			
18	0	0	0	290	15	25			
19	0	0	0	290	15	25			
20	0	0	0	290	15	25			

Abbreviations: W, wheat flour; R, rice bran; B, buckwheat bran; Y_1_, baking strength of dough; Y_2_, water absorption capacity; Y_3_, organoleptic indices.

**Table 3:** Results of Experimental Studies.

No.	Encoded Values			Natural Values			Experimental Values		
	x_1_	x_2_	x_3_	W, g	R, g	B, g	Y_1_	Y_2_	Y_3_
1	-	-	-	280	10	20	352	60.1	18
2	-	-	+	280	10	30	320	59.9	16
3	-	+	-	280	20	20	305	58.2	15
4	-	+	+	280	20	30	295	58.8	14
5	+	-	-	300	10	20	310	60.4	15
6	+	-	+	300	10	30	339	60.5	16
7	+	+	-	300	20	20	198	59.9	15
8	+	+	+	300	20	30	300	60.8	17
9	−1.68	0	0	306.82	15	25	320	61.3	14
10	+1.68	0	0	273.18	15	25	283	60.4	16
11	0	−1.68	0	290	23.41	25	275	57.8	12
12	0	+1.68	0	290	6.59	25	335	61.4	14
13	0	0	−1.68	290	15	33.41	306	61.1	17
14	0	0	+1.68	290	15	16.59	325	59.1	14
15	0	0	0	290	15	25	275	61.1	15
16	0	0	0	290	15	25	280	61.0	15
17	0	0	0	290	15	25	278	61.2	15
18	0	0	0	290	15	25	282	61.1	15
19	0	0	0	290	15	25	277	61.1	15
20	0	0	0	290	15	25	280	61.2	15

Abbreviations: W, wheat flour; R, rice bran; B, buckwheat bran; Y_1_, baking strength of dough; Y_2_, water absorption capacity; Y_3_, organoleptic indices.

**Table 4:** Technological parameters of bread dough preparation.

Name of Physicochemical Indicators of the Process	Meaning of Dough Values for Bread		
	Control (Without Additives)	With the Application of Rice and Buckwheat Brans in the Ratio of 5:10	
		In the Dry State	In the Form of Fermented Brew
Humidity, %	47.0 ± 1.0		
Temperature, °C			
- initial	29 ± 1 ^a^	30 ± 1 ^a^	29 ± 1 ^a^
- final	31 ± 1 ^a^	31 ± 1 ^a^	30 ± 1 ^a^
Acidity, deg			
- initial	2.3 ± 0.5 ^a^	3.4 ± 0.5 ^b^	8.2 ± 1.0 ^c^
- final	2.6 ± 0.5 ^a^	4.5 ± 0.5 ^b^	9.0 ± 1.0 ^c^
Fermentation duration, min	60 ± 5		
Volume increase, %	77.4 ± 0.1 ^a^	90 ± 0.1 ^b^	100 ± 0.1 ^c^
Lifting force, min	4 ± 1 ^a^	10 ± 1 ^b^	7 ± 1 ^c^
Proofing time, min	55 ± 5 ^a^	55 ± 5 ^a^	45 ± 5 ^b^

^a–c^ = means ± SD within the same line with different lowercase superscript letters being significantly different (*p* ≤ 0.05).

**Table 5:** Effect of rice and buckwheat bran application method on physicochemical parameters of bread.

Name of Process Indicators	Meaning of Bread Indicators		
	Control (Without Additives)	With Rice and Buckwheat Brans in the Ratio of 5:10	
		In the Dry State	In the Form of Fermented Brew
Humidity, %			
- whole bread	41.2 ± 0.5 ^a^	41.3 ± 0.5 ^a^	42.4 ± 0.5 ^b^
- crumb	47.0 ± 0.5 ^a^	48.0 ± 0.5 ^b^	48.5 ± 0.5 ^c^
Acidity, grad	1.8 ± 0.1 ^a^	2.8 ± 0.1 ^b^	6.0 ± 0.5 ^c^
Porosity, %	78 ± 2 ^a^	78 ± 2 ^a^	73 ± 2 ^b^
Specific volume, cm^3^/g	2.96 ± 0.1 ^a^	2.96 ± 0.1 ^a^	2.51 ± 0.1 ^b^
Alcohol content, % on dry substances	0.48 ± 0.1 ^a^	0.51 ± 0.1 ^b^	0.96 ± 0.1 ^c^
Volatile acid content			
- hail	0.70 ± 0.1 ^a^	0.8 ± 0.1 ^a^	2.05 ± 0.1 ^b^
- % of total acidity	38.9 ± 0.1 ^a^	28.6 ± 0.1 ^b^	34.2 ± 0.1 ^c^
Sugar content			
- % on dry substances	4.32 ± 0.1 ^a^	4.14 ± 0.1 ^a^	3.10 ± 0.1 ^c^
g per 100 g of product	2.54 ± 0.1 ^a^	2.43 ± 0.1 ^a^	1.79 ± 0.1 ^b^

^a–c^ = means ± SD within the same line with different lowercase superscript letters being significantly different (*p* ≤ 0.05).

**Table 6:** Changes in moisture content and structural–mechanical properties of bread crumb during storage.

Name of Process Indicators	Meaning of Bread Indicators		
	Control (Without Additives)	With Rice and Buckwheat Brans in the Ratio of 5:10	
		In the Dry State	In the Form of Fermented Brew
Crumb moisture, %			
after 24 h	47.0 ± 1.0 ^a^	48.1 ± 1.0 ^b^	48.6 ± 1.0 ^c^
after 48 h	46.2 ± 1.0 ^a^	48.0 ± 1.0 ^b^	48.5 ± 1.0 ^c^
after 72 h	46.0 ± 1.0 ^a^	47.7 ± 1.0 ^b^	48.2 ± 1.0 ^c^
Crumbliness, %			
after 24 h	4.15 ± 0.10 ^a^	1.88 ± 0.10 ^b^	1.66 ± 0.10 ^c^
after 48 h	4.48 ± 0.10 ^a^	3.43 ± 0.10 ^b^	3.83 ± 0.10 ^c^
after 72 h	7.15 ± 0.11 ^a^	6.10 ± 0.11 ^b^	4.38 ± 0.11 ^c^
Physical properties of the crumb: hardness index, G/[(g/cm^3^)%]			
after 24 h	26.1 ± 0.1 ^a^	26.2 ± 0.1 ^a^	25.2 ± 0.1 ^b^
after 48 h	33.9 ± 0.2 ^a^	32.0 ± 0.1 ^b^	33.5 ± 0.1 ^c^
after 72 h	37.3 ± 0.2 ^a^	34.1 ± 0.1 ^b^	39.8 ± 0.2 ^c^

^a–c^ = means ± SD within the same line with different lowercase superscript letters being significantly different (*p* ≤ 0.05).

**Table 7:** Chemical composition and nutritional value of bread.

Name of Indicators	Value of Indicators (g/100 g) of Bread	
	Control (Without Additives)	Using Rice and Buckwheat Brans in the Form of Fermented Brew
Protein, g	8.17 ± 0.03 ^a^	12.97 ± 0.05 ^b^
Fats, g	0.99 ± 0.02 ^a^	6.0 ± 0.07 ^b^
Carbohydrates, g	44.35 ± 0.68 ^a^	33.46 ± 0.24 ^b^
Dietary fiber, g	2.63 ± 0.03 ^a^	2.85 ± 0.03 ^b^
Vitamins. mg:		
E	1.3 ± 0.03 ^a^	0.768 ± 0.05 ^b^
PP (niacin)	0.42 ± 0.01 ^a^	0.279 ± 0.056 ^b^
B2 (riboflavin)	0.03 ± 0.01 ^a^	0.050 ± 0.021 ^b^
B5 (pantothenic acid)	-	0.074 ± 0.013
B6 (pyridoxine)	-	0.046 ± 0.009
Mineral substances. mg:		
Iron	0.95 ± 0.08 ^a^	5.47 ± 0.05 ^b^
Sodium	381.71 ± 2.4 ^a^	355.19 ± 2.1 ^b^
Magnesium	12.54 ± 0.06 ^a^	75.78 ± 0.51 ^b^
Calcium	17.35 ± 0.25 ^a^	30.21 ± 0.06 ^b^
Potassium	94.84 ± 1.60 ^a^	268.93 ± 0.97 ^b^
Phosphorus	67.48 ± 1.53 ^a^	218.66 ± 1.05 ^b^
Energy value. kcal	219.0 ± 1.50 ^a^	239.72 ± 1.50 ^b^

^a–b^ = means ± SD within the same line with different lowercase superscript letters being significantly different (*p* ≤ 0.05).

**Table 8:** Satisfaction of the daily requirement of an adult in nutrients when eating 250 g of bread.

Name of Main Nutritional Substances	Daily Energy and Nutrient Requirements for Adults	Value of Indicators of Satisfaction of Daily Requirement in Nutrients When Consuming 250 g of Bread			
		Control (Without Additives)		Using Rice and Buckwheat Brans in the Form of Fermented Brew	
		g	%	g	%
Protein. g	104	20.4	19.6	32.4	31.2
Fats. g	100	2.5	2.5	15	15
Carbohydrates. g	360	110.9	30.8	83.6	23.2
Dietary fiber. g	38.6	6.6	17	7.1	18.4
Vitamins. mg/:					
E	26	3.3	12.7	1.9	7.3
PP (niacin)	18.6	1.0	5.4	0.7	3.8
B2 (riboflavin)	1.7	0.1	5.8	0.1	5.8
B5 (pantothenic acid)	10	-	-	0.2	2
B6 (pyridoxine)	2	-	-	0.1	5
Minerals. mg/:					
Iron	25	2.3	9.2	13.7	54.8
Sodium	2000	954.3	47.7	888.0	44.4
Magnesium	400	31.35	7.8	189.5	47.4
Calcium	957	43.4	4.5	75.5	7.9
Potassium	2500	237.1	9.5	672.3	26.9
Phosphorus	1000	168.7	16.87	546.7	54.7

## Data Availability

The original contributions presented in the study are included in the article, further inquiries can be directed to the corresponding authors.
